# Thought to Be Extinct, but Still Alive Today: The Miocene Genus *Primascena* Klimaszewsi, 1997 (Hemiptera: Psyllidae) in the Light of Two Extant Species from Brazil

**DOI:** 10.3390/insects15060382

**Published:** 2024-05-23

**Authors:** Daniel Burckhardt, Dalva L. Queiroz

**Affiliations:** 1 Naturhistorisches Museum, Augustinergasse 2, 4052 Basel, Switzerland; 2 Embrapa Florestas, Empresa Brasileira de Pesquisa Agropecuária, Km 111, Bairro Guaraituba, Colombo 83411-000, PR, Brazil; dalva.queiroz@embrapa.br

**Keywords:** Sternorrhyncha, fossils, taxonomy, neotropical, Polygonaceae, *Ruprechtia*

## Abstract

**Simple Summary:**

The psyllid genus *Primascena* was erected for a fossil species from Dominican amber, *P. subita*. As the single known specimen of this species is a partly damaged male, the description was quite incomplete. Recently, two undescribed species were discovered in Brazil (Mato Grosso do Sul) that are ascribed to this genus. The extant species allow the description of the previously unknown female and immature, and to complete information on the morphology of the male. A cladistic morphological analysis confirms the position of the genus in the subfamily Aphalaroidinae and supports the monophyly of the fossil and the two extant species. The extant *Primascena* species develop on plants of the Polygonaceae: Eriogonoideae, an unusual psyllid host, shared by only one other species of the same subfamily. Aphalaroidinae is most species-rich in the neotropics, with nine out of 13 genera being restricted to South America.

**Abstract:**

Fossils can document the morphological diversification through time and date lineages, providing relevant characters are preserved. *Primascena* Klimaszewsi, 1997 was erected for *P. subita* Klimaszewsi, 1997 on the basis of a single, partly damaged male from Dominican amber. Originally assigned to Rhinocolidae: Paurocephalinae, the genus was subsequently transferred to Psyllidae: Aphalaroidinae. Recently, two undescribed species resembling the fossil species were discovered in Brazil (Mato Grosso do Sul), allowing a detailed morphological study of adults and immatures. Based on the morphological study, a revised diagnosis of the genus is provided, including the previously unknown female and fifth instar immatures. *Primascena subita* is redescribed and *P. empsycha* n. spec. and *P. ruprechtiae* n. spec. are formally described and illustrated. An identification key is provided for the species of *Primascena*. A cladistic morphological analysis supports the placement of the two new species in *Primascena*, and of this genus in the Aphalaroidinae. It is sister to all but *Aphalaroida*, though with little support. The two Brazilian species develop on *Ruprechtia* spp. (Polygonaceae: Eriogonoideae), an unusual psyllid host. Immatures of *P. ruprechtiae* are free-living on the lower leaf face and do not induce galls.

## 1. Introduction

Fossils illustrate the diversification of morphological structures through time and may help dating the age of lineages, providing the diagnostic characters are preserved. This is, however, often not the case, making fossils difficult to place in a phylogeny/classification of extant taxa. The jumping plant lice or psyllids (Hemiptera: Sternorrhyncha: Psylloidea) serve as an example. In representatives of this taxon, phylogenetically relevant characters are often difficult to observe (e.g., chaetotaxy of immatures), and morphological homoplasy is widespread (e.g., presence/absence of a genal processes) [1,2]. Psyllids constitute arguably the least known superfamily of Sternorrhyncha, with slightly over 4000 described species worldwide and at least as many undescribed species [3,4,5]. Psyllids are phloem feeders with generally narrow host ranges. Often, hosts of related psyllid taxa are restricted to a single plant family or order. The majority of host plants are eudicots and, to a much lesser extent, magnoliids. Only a few psyllid species develop on monocots, and even fewer on conifers [6,7,8,9].

Fossils of jumping plant lice are rare and completely absent from the Coniacian (late Upper Cretaceous) to the Ypresian (early Eocene), a period of over 40 million years coinciding almost exactly with the angiosperm radiation during the Upper Cretaceous and Paleocene. Ouvrard, et al. [1] suggested that the explosion of flowering plant diversity has drastically influenced the evolution of Psylloidea with the appearance of modified morphological and biological traits in modern psyllids. The oldest true psyllids, i.e., with modified hind legs, appear in the Lutetian (Middle Eocene), representing the extinct tribe Paleopsylloidini† (Aphalaridae, Aphalarinae), with eight Eocene/Oligocene genera and one extinct monotypic genus of the subfamily Rhinocolinae (Aphalaridae) [1,4]. While the Eocene/Oligocene psyllid genera are all extinct members of the family Aphalaridae, the known fauna of Miocene psyllids is more diverse at the family and genus levels, and is similar to the present day fauna [10]. This is documented for the inclusions in Dominican amber (mid-Miocene, 20–15 Ma [11]) [12,13]. Currently, 16 genera have been recorded from Dominican amber from the Aphalaridae (2 genera), Liviidae (3 genera), Psyllidae (7 genera), and Triozidae (4 genera), but many more taxa remain to be described [14]. Nine of the sixteen genera include also extant species, but seven are known only from fossils. The seven fossil-only genera are all monotypic, each based on a single specimen, and their descriptions are not diagnostic [12,13].

*Prismascena* Klimaszewski, 1997 is one of these genera. Klimaszewski [13] referred the genus to the Paurocephalinae for the alleged lack of genal processes and metabasitarsal spurs. In contrast, Burckhardt and Mifsud [15] examined the holotype and noticed genal processes and metabasitarsal spurs. They concluded that the genus probably belongs to the Aphalaroidinae, a putatively monophyletic subfamily of Psyllidae [4,16,17] with a surprisingly broad host range (Table 1). The subfamily Aphalaroidinae was erected by Vondráček [18] for *Aphalaroida* and members of the unrelated Paurocephalini on the basis of the presence of a pterostigma in the forewing and the lack of following three characters: (1) genal processes, (2) cross-vein between or partial fusion of veins Rs and M of the forewing, and (3) posterior lobes on the male proctiger. Within Psylloidea, these characters represent homoplasies and are of little phylogenetic significance. Burckhardt [16] redefined the subfamily to comprise eight genera restricted to the Americas, using characters of adults and immatures. Later, Burckhardt [9] added another five genera, including one each from Africa and Asia, respectively. In the most comprehensive molecular analysis of Psylloidea by Percy, et al. [17], four genera of Aphalaroidinae recognized by Burckhardt and Ouvrard [19] were included, of which three formed a strongly supported clade while one, the genus *Telmapsylla* Hodkinson, grouped with the Ciriacreminae to which it was transferred by Burckhardt, et al. [4]. In the most recent classification of Psylloidea [4] based on molecular and morphological evidence, the Aphalaroidinae comprises 13 genera, including *Primascena*, with around 100 described species [5].

On a recent field trip to the Pantanal (Brazil), we discovered two undescribed species not belonging to any of the genera known from Brazil. Both species were collected on *Ruprechtia* (Polygonaceae), an uncommon psyllid host family [20]. Detailed morphological studies suggest that the two new species from Brazil are most closely related to *P. subita*, the type species of *Primascena*. Here, we describe the two new species, redefine *Primascena* and redescribe *P. subita*, and analyze the phylogenetic relationships within the Aphalaroidinae with a cladistic analysis using morphological characters. The aim of the cladistic analysis is to test the monophyly of *Primascena* and examine the phylogenetic relationships of *Primascena* within the genus and the subfamily.

**Table 1:** Recognized genera in Aphalaroidinae with number of described species, host plants (confirmed and likely, with number of associated species), and general distribution [5] (unpublished NHMB data).

Psyllid Genus	Number of Species	Host Plants	Distribution
*Aphalaroida* Crawford [21]	9	Fabaceae (8)	North America
*Baccharopelma* Burckhardt, et al. [22]	6	Asteraceae (5)	South America
*Connectopelma* Šulc [23]	6	Rhamnaceae (6)	South America
*Ehrendorferiana* Burckhardt [9]	2	Cupressaceae (2)	South America
*Freysuila* Aleman [24]	3	Fabaceae (2), Santalaceae (1)	North and South America
*Pachyparia* Loginova [25]	1	Fabaceae (1)	Africa
*Panisopelma* Enderlein [26]	12	Zygophyllaceae (10)	South America
*Primascena* Klimaszewski [13]	3 (including 1 fossil)	Polygonaceae (2)	South America
*Prosopidopsylla* Burckhardt [16]	5	Fabaceae (5)	South America
*Russelliana* Tuthill [27]	43	Asteraceae (4), Fabaceae (12), Polygonaceae (1), Rosaceae (2), Solanaceae (16), Verbenaceae (5), polyphagous (1)	South America
*Sphinia* Blanchard [28]	6	Euphorbiaceae (4), Rosaceae (2)	South America
*Yangus* Fang [29]	3	Fabaceae (3)	Asia
*Zonopelma* Burckhardt [16]	2	Misodendraceae (2)	South America

## 2. Materials and Methods

### 2.1. Material Examined

Material was examined or is cited from following institutions: American Museum of Natural History, New York, NY, USA (AMNH); Naturhistorisches Museum, Basel, Switzerland (NHMB); Muséum d’histoire naturelle, Geneva, Switzerland (MHNG); Coleção Entomológica Padre Jesus Santiago Moure, Centro Politécnico, Universidade Federal do Paraná, Curitiba, PR, Brazil (UFPR). The field collections of psyllids in Brazil were carried out with permit CNPq, IBAMA/SISBIO (71632-3 41169-12).

A dissecting microscope and a compound microscope with brightfield and darkfield, as well as interference contrast, were used for the morphological observations. The morphological terminology follows mostly Bastin, et al. [30], and that of the thorax Ouvrard, et al. [31] and Drohojowska [32]. Measurements were taken as follows: adult body length from specimens preserved in 70% ethanol, measuring the distance between fore margin of head and tip of forewings when folded over body; the other measurements were taken from slide-mounted specimens. The measurements and ratios are provided as ranges. The nomenclature and classification of Psylloidea follows Ouvrard [5] and Burckhardt, et al. [4], respectively, and that of the plants accords with POWO [33]. Here, we adopt the “Diagnosibility Version of the Phylogenetic Species Concept” described by Zachos [34] which is based, among others, on Nelson and Platnick [35], who define a species with a unique diagnosable combination of characters.

### 2.2. Taxon and Character Selection

Thirty species were included, representing all 13 genera recognized by Burckhardt, et al. [4] by one to three species each. Whenever possible, species were selected from which both sexes and immatures are known. As outgroup and for rooting the tree, *Acizzia hollisi* Burckhardt, 1981 [36] was chosen, a member of the subfamily Acizziinae that, among others, belongs to the sister-clade of Aphalaroidinae [17].

A total of 40 morphological characters were selected for the cladistic analysis: 30 from adults and 10 from the last instar immatures. A total of 17 characters are binary and 23 are multistate. Missing data were coded as “?”. All characters were treated as unordered. The morphological data were obtained from material deposited in the NHMB. The character matrix was assembled with WinClada v1.00.80 [37] (Supplementary File S1).

### 2.3. Phylogenetic Analysis

**Figure 1:**
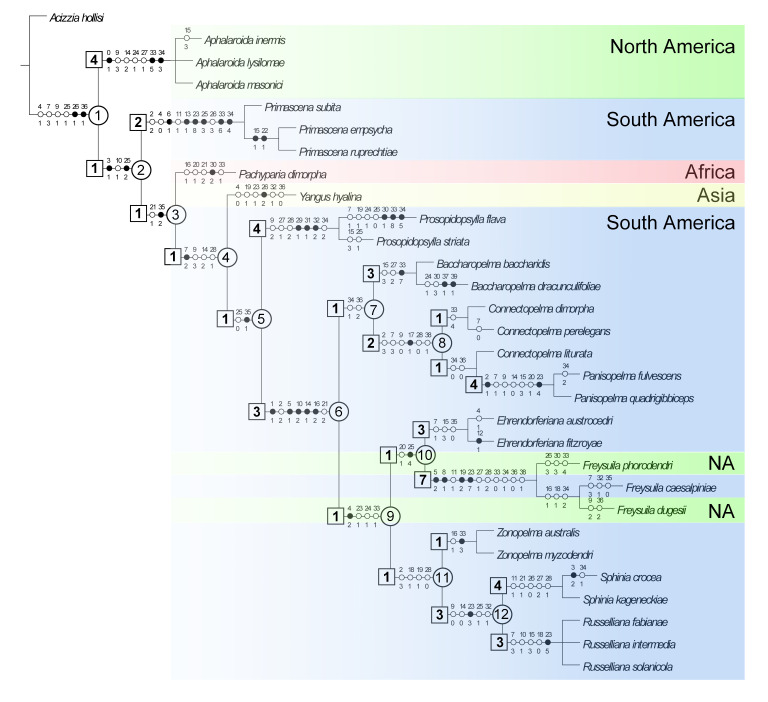
Phylogenetic relationships among the known genera of Aphalaroidinae (outgroup = *Acizzia hollisi*). For *Aphalaroida*, *Baccharopelma*, *Connectopelma*, *Panisopelma*, *Prosopidopsylla*, *Russelliana*, *Sphinia*, and *Yangus*, only a selection of the known species is included. The single most parsimonious tree was obtained by cladistic analysis of unweighted characters. The character state changes are shown for each node using the ‘Fast optimization’ option of WinClada. Synapomorphies are represented with black circles and homoplasies with white circles. Bremer support values are indicated in squares at the base of branches and the numbers of nodes are provided in circles. NA = North America.

A parsimony analysis (unweighted) was performed with TNT v.1.5 [38] using the ‘Traditional search’ method with the following settings: random seed = 0, replications = 1000, swapping algorithm = TBR with 1000 trees to save per replication. The matrix was analyzed with the options ‘equal weights’ and ‘implied weights’. The character state changes are shown for each node (Figure 1), using the ‘Fast optimization’ option of WinClada. The ‘fast optimization’ places the change on the tree as soon as possible (sometimes called accelerated transformation or ACCTRAN) [39].

List of Characters
Adult

0. Glandular hairs on body integument: absent (0); present (1).
1. Vertex: subrectangular (0); trapezoidal (1).
2. Vertex: flat (0); with flat anterior lobes (1); weakly bulged anteriorly (2); with large anterior tubercles (3).
3. Genal processes: absent (0); present, shorter than vertex (1); present, longer than vertex, (2).
4. Preocular sclerite: absent (0); present as small sclerite, narrower than one fifth of the eye width (1); present as large sclerite, wider than one quarter of the eye width (2).
5. Eyes: hemispherical, adpressed to head (0); weakly stalked, posterior margin of head not indented between eye and lateral ocellus (1); strongly stalked, posterior margin of head indented between eye and lateral ocellus (2).
6. Sensory plates on antennal segment III: absent (0); present (1).
7. Antennal rhinaria: without a wreath of spines (0); with a partial wreath of spines on all segments (1); with a complete wreath of spines on all segments (2); on some segments with complete or partial wreath of spines (3).
8. Rostrum: short, stout or moderately long but only tip of apical segment visible in lateral view (0); long, slender, entire apical segment and part of subapical segment visible in lateral view (1).
9. Pronotum: flat (0); with two tubercules on either side (1); with a pit on either side (2); with two pits on either side (3).
10. Propleurites: broader than high, epimeron and episternum subequal (0); higher than broad, epimeron and episternum subequal (1); narrow, episternum much smaller than epimeron (2).
11. Tubercle on metapostnotum: flat (0); large, strongly produced (1).
12. Genual spine: absent (0); present (1).
13. Metatibial and metabasitarsal spurs: strongly sclerotized (0); hardly sclerotized (1).
14. Metatibia bearing: a posteriorly open crown of < 8 evenly spaced apical spurs (0); a posteriorly open crown of >7 densely spaced apical spurs (1); distinctly grouped apical spurs (2).
15. Metabasitarsus: with 2 lateral spurs (0); with 1 lateral spur (1); with 1 microscopical lateral spur (2); without lateral spurs (3).
16. Length of forewing vein R relative to that of M+Cu: shorter (0); as long as (1); longer (2).
17. Forewing: Rs and M separate (0); Rs and M touching in a point or partially fused (1); with r-m crossvein (2).
18. Forewing vein M: longer than its branches (0); shorter than its branches (1).
19. Forewing cell cu_1_: short, about 1.5 times as high as long (0); longer than high (1); about twice as high as long (2).
20. Hindwing costal setae: not grouped (0); grouped (1).
21. Hindwing vein R+M+Cu: trifurcating into R, M and Cu (0); bifurcating into R and M+Cu (1); bifurcating into R+M and Cu (2).

Terminalia

22. Male subgenital plate: subglobular (0); distinctly elongate (1).
23. Paramere: lamellar with antero-apical or apical sclerotized tooth (0); slender, digitiform (1); lamellar, with forward-directed tooth, and inner tooth at apical margin (2); base robust, apex slender, backwards directed (3); complex, sickle-shaped (4); short, with forward-directed hook (5); short, rectangular, sometimes with antero-apical process (6); lamellar/spiniform with inner sclerotized process/tooth in basal half (7); oval or rectangular with inner process (8).
24. Distal segment of aedeagus: without lateral lobes (0); with lateral lobes (1).
25. Distal segment of aedeagus: with small, oval apical inflation (0); with large, irregularly triangular apical inflation (1); with large apical inflation divided into ventral and dorsal parts (2); with moderately sized to large apical inflation incised dorso-basally (3); tubular with appendages (4).
26. Sclerotized end tube of ductus ejaculatorius: small, shorter than 5 times its diameter (0); medium sized, 5–10 times as long as its diameter and less than a fifth of the length of the distal segment of aedeagus (1); large, a quarter of the length of the distal segment of aedeagus (2); very large, longer than a third of the length of the distal segment of aedeagus (3).
27. Female proctiger with dorsal margin: straight or curved (0); with a slight depression distal to circumanal ring (1); strongly bent down distal to circumanal ring (2).
28. Female subgenial plate: long, in lateral view longer than high, lacking glandular setae (0); short, in lateral view as long as high, lacking glandular setae (1); short, in lateral view as long as high, with glandular setae (2).
29. Brush-like combs of fringing hairs on distal abdominal sternites in female: absent (0); present (1).

Fifth instar immature

30. Antenna: 7-segmented (0); 5-segmented (1); 3-segmented (2); 8-segmented (3).
31. Humeral lobes: absent (0); present (1).
32. Thoracic tergites: small, covering less than half of the surface of meso and metanotum (0); medium-sized, covering more than half of the surface of meso and metanotum but leaving membranous areas (1); large, almost completely covering meso and metanotum (2).
33. Body margin and dorsum of immature: with long capitate setae (0); with short or no marginal setae (1); with diamond-shaped setae on margin of wing pads (2); with a few marginal capitate setae on abdomen, otherwise without setae (3); with bristle-like setae (4); with marginal rod setae on caudal plate, without dorsal setae (5); with capitate and short funnel-shaped setae (6); with simple setae (7); with club-shaped setae (8).
34. Immature forewing pads: with no or few long dorsal or marginal simple setae (0); with moderately long simple setae (1); with long capitate setae (2); with marginal long rod setae (3); with long marginal and funnel-shaped dorsal setae (4); with diamond or club-shaped setae (5)
35. Pedicel of tarsal arolium: as long as claws (0); longer than claws (1); shorter than claws (2).
36. Anus: ventral, near hind margin, distance from anus to hind margin of caudal plate less than maximum length of circumanal ring (0); ventral, distant from hind margin, distance from anus to hind margin of caudal plate more than maximum length of circumanal ring (1); terminal (2).
37. Circumanal ring: unicellular (0); multicellular (1).
38. Circumanal ring: small, narrower than a third width of terminal ventrite (0); large, wider than three quarters width of terminal ventrite (1).
39. Pores of the circumanal ring: narrow, elongate (0); circular (1).

## 3. Results

### 3.1. Phylogeny

The ‘Traditional search’ with TNT using equal character weights calculated a single most parsimonious tree (Figure 1) of 154 steps with a consistency index CI = 53 and a retention index RI = 76. The cladogram is fully resolved except for the two genera, *Aphalaroida* Crawford and *Russelliana* Tuthill, which each have three unresolved species. When using implied weights (K = 3.0000) [38], the analysis resulted in two most parsimonious trees whose consensus tree had a similar topology but was only partly resolved. For this reason, we do not further discuss the latter analysis.

Monophyly and internal relationships of *Primascena*. The monophyly of *Primascena* is supported by six synapomorphies and four homoplasies with a Bremer clade support = 2. The synapomorphies are as follows: sensory plates on antennal segment III present (6-1), metatibial and metabasitarsal spurs weakly or hardly sclerotized (13-1), paramere oval or rectangular with inner process (23-8), distal segment of aedeagus with moderately sized to large apical inflation incised dorso-basally (25-3), body margin and dorsum of immature with capitate and short funnel-like setae (33-6), and immature forewing pads with long marginal and funnel-shaped dorsal setae (34-4); the homoplasies are: vertex weakly bulged anteriorly (2-2), preocular sclerite absent (4-0), tubercle on metapostnotum large (11-1), and sclerotized end tube of ductus ejaculatorius very large (26-3). Within *Primascena* the fossil *P. subita* constitutes the sister taxon of the two extant species grouped together by two synapomorphies: metabasitarsus with one lateral spur (15-1), and male subgenital plate distinctly elongate (22-1).

Phylogenetic relationships within *Primascena* and within the Aphalaroidinae. Node 1 (Figure 1) splits into the North American *Aphalaroida* and node 2. The monophyly of the former is well supported by three synapomorphies and four homoplasies with a Bremer support = 4. Node 2 is supported by two synapomorphies and one homoplasy with a Bremer support = 1. The synapomorphies are as follows: genal processes present, shorter than vertex (3-1), distal segment of aedeagus with large apical inflation divided into a ventral and dorsal part (25-2); the homoplasy is propleurites higher than broad, epimeron, and episternum subequal (10-1). Node 2 splits into the strongly supported *Primascena* (see above) and the weakly supported node 3 (1 synapomorphy (36-2) and 1 homoplasy (21-2) with Bremer support = 1) which includes the remainder of genera. The supports of nodes 3 and 4 are weak, each with only one synapomorphy and a Bremer support = 1. The monotypic African *Pachyparia* is sister group of node 4, and *Yangus* with four described Asian species [5] and several undescribed Afrotropical species (unpublished NHMB data) is the sister group of node 5. Members of node 5 are restricted to America [5]. Node 5 includes the well-supported *Prosopidopsylla* (3 synapomorphies and 4 homoplasies with Bremer support = 4) and the well-supported node 6 (5 synapomorphies and 2 homoplasies with Bremer support = 3). While six (*Baccharopelma* Burckhardt, Espírito-Santo, Fernandes & Malenovský, *Ehrendorferiana* Burckhardt, *Freysuila* Aleman, *Panisopelma* Enderlein, *Russelliana* Tuthill, *Sphinia* Blanchard) of the eight genera included in node 6 have Bremer supports of 3 or more, *Zonopelma* Burckhardt has a Bremer support of only 1 and *Connectopelma* Šulc is paraphyletic. Also, the relationships between the genera of node 6 are only weakly supported.

### 3.2. Taxonomy

#### 3.2.1. Subfamily: Aphalaroidinae Vondráček, 1963: 277 [18]. Type: genus. *Aphalaroida* Crawford

Subfamily: Arepuninae White & Hodkinson, 1985: 227 [28]. Type: genus. *Arepuna* Tuthill. Synonymised by Burckhardt, 1987: 315 [16].

Comments. Morphological diagnoses of the subfamily were provided by Burckhardt [16], Burckhardt and Ouvrard [19], and Burckhardt et al. [4].

#### 3.2.2. Genus: *Primascena* Klimaszewsi, 1997 [13]

*Primascena* Klimaszewsi, 1998: 20. Type: species. *Primascena subita* Klimaszewsi, by original designation and monotypy.

Diagnosis. Adult. Body small, 1.4–1.9 mm long. Vertex 0.6–0.7 times as long as wide. Genae forming conical, apically subacute processes. Preocular sclerite absent. Compound eyes hemispherical, adpressed to head. Antennal length 1.0–1.3 times head width, segment III with apical disc-shaped sensorium. Propleurites as high as wide, with anterior margin of mesosternum weakly concave; pleurosternal suture not visible; katepisternum indistinct, only weakly produced anteriorly; basisternum indistinctly triangular; in ventral view, tubercle of trochantin anteriad of mesocoxa weakly developed; precoxale forming obtuse angle. Metatibia with a posteriorly open crown of 6–8 weakly or hardly sclerotized apical spurs. Metabasitarsus bearing one outer, or two weakly or hardly sclerotized lateral spur. Forewing oblong–oval, 2.7–3.2 times as long as head width, 2.4–2.5 times as long as wide; pterostigma ending above bifurcation of M; veins Rs and M_1+2_ not fused or connected by cross-vein; vein M much longer than its branches; cells m_1_ and cu_1_ moderately large. Hindwing with costal setae not grouped; vein R+M+Cu indistinctly bifurcating into veins R and M, base of Cu indistinct. Male proctiger narrowly tubular. Paramere, in lateral view, lamellar, shorter than proctiger. Distal segment of aedeagus lacking lateral processes, bearing a moderately sized to large apical inflation which is incised dorso-basally. Female terminalia long, cuneate. Fifth instar immature. Body in dorsal view subcircular, 1.0–1.2 times as long as wide. Forewing pad small, oval; beset with long and short marginal, and short dorsal funnel-shaped setae. Hindwing pad with moderately long marginal and short dorsal funnel-shaped setae. Caudal plate evenly rounded posteriorly, with long marginal capitate setae and short dorsal funnel-shaped setae.

**Figure 2:**
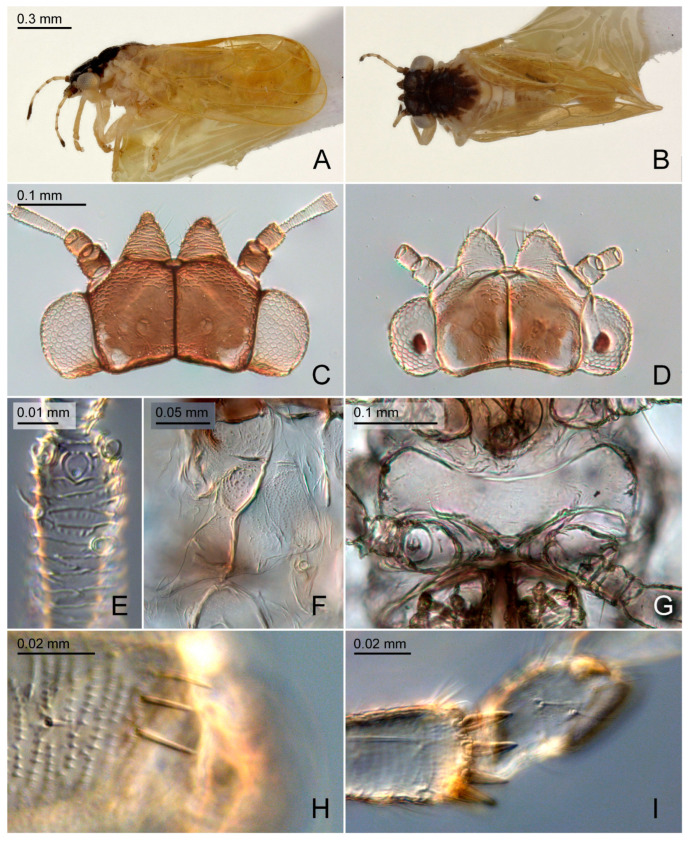
*Primascena* spp.: (**A**–**C**) *P. empsycha*; (**D**–**I**) *P. ruprechtiae*. (**A**) Habitus, dorsal view; (**B**) habitus, lateral view; (**C**,**D**) head, dorsal view; (**E**) apical half of antennal segment III; (**F**) propleurites, dorsad up, anteriad left; (**G**) mesosternum; (**H**) apex of metafemur; (**I**) apex of metatibia and metabasitarsus.

**Figure 3:**
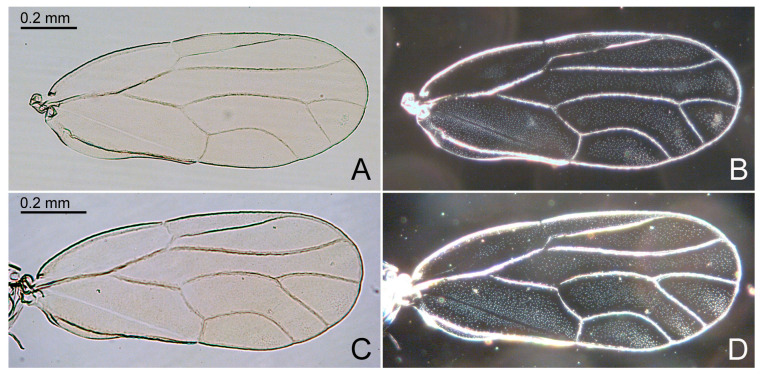
*Primascena* spp., forewing: (**A**,**B**) *P. empsycha*; (**C**,**D**) *P. ruprechtiae*. (**A**,**C**) Forewing: shape and venation (brightfield); (**B**,**D**) forewing: distribution of surface spinules (darkfield).

**Figure 4:**
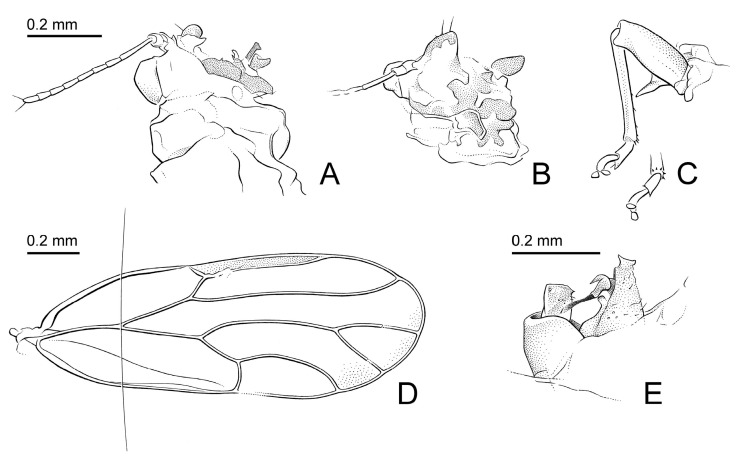
*Primascena subita* Klimaszewski, 1997; holotype ♂. (**A**) Head, antenna, and pronotum, dorsal view; (**B**) head and basal segments of antenna, ventral view; (**C**) hind leg, outer face and tip of leg, inner face; (**D**) forewing; (**E**) male terminalia (drawings by Armin Coray).

**Figure 5:**
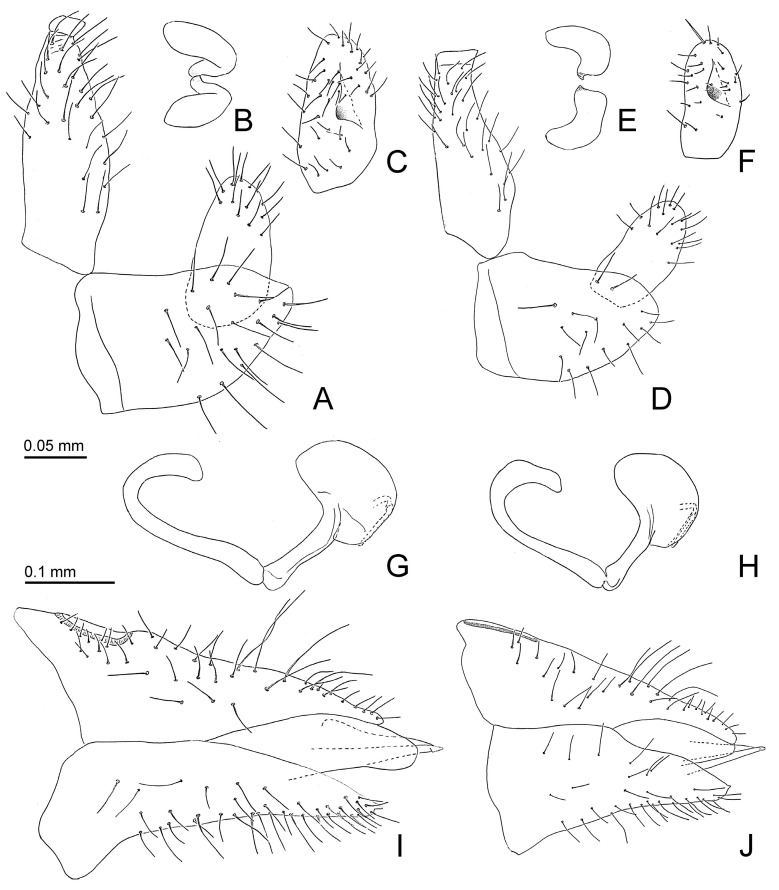
*Primascena* spp., terminalia. (**A**–**C**,**G**,**I**) *P. empsycha*; (**D**–**F**,**H**,**J**) *P. ruprechtiae*. (**A**,**D**) Male terminalia, in lateral view; (**B**,**E**) parameres, dorsal view, left anteriad, right posteriad; (**C**,**F**) parameres, inner face, in lateral view; (**G**,**H**) aedeagus, lateral view; (**I**,**J**) female terminalia, in lateral view.

**Figure 6:**
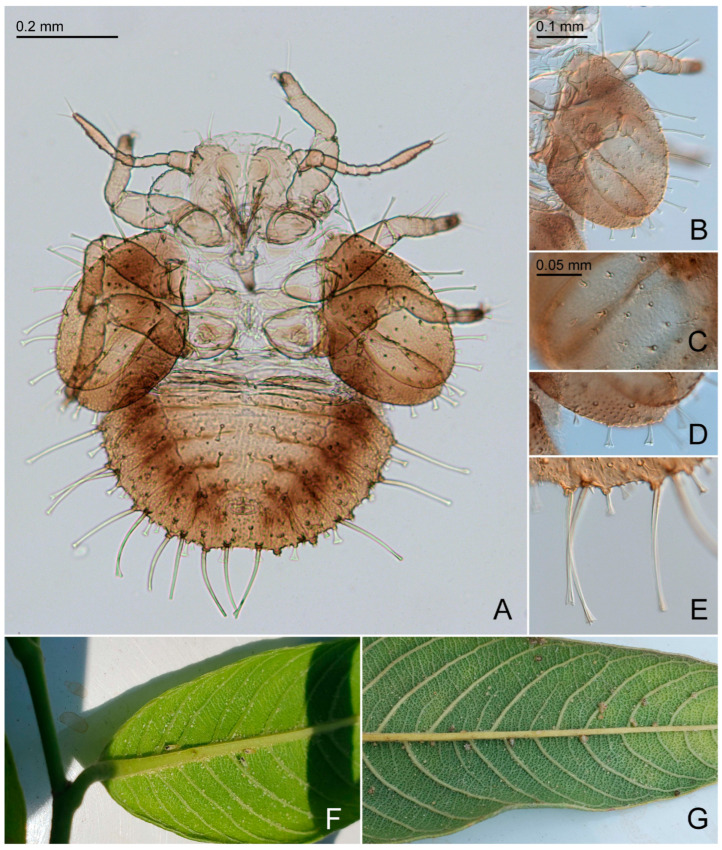
*Primascena ruprechtiae*, immatures. (**A**) Habitus, dorsal view; (**B**) wing pads, dorsal view; (**C**) disca of forewing pad, dorsal view; (**D**) margin of hindwing pad, dorsal view; (**E**) margin of caudal plate, dorsal view; (**F**) adults and eggs on abaxial leaf surface; (**G**) immatures on abaxial leaf surface.

Additional characters. Adult. Body small (Figure 2A,B), length (including wings) 1.4–1.9 mm. Head, thoracic notum, and wings lacking glandular setae. Head about as wide as thorax, inclined not more than 30° from longitudinal body axis. Vertex (Figure 2C,D) flat except for weakly indented foveae, 0.6–0.7 times as long as wide; sparsely beset with short setae, covered with imbricate microsculpture around the margins, smooth on disc; median suture fully developed; posterior margin of vertex weakly concave; anteriorly delimited by transverse indistinct edge, not forming tubercles antero-medially. Genae forming conical, apically subacute processes, 0.4–0.7 times as long as vertex along mid-line, dorsal face lying in a plane below that of vertex. Frons small, trapezoidal, mostly covered by median ocellus. Preocular sclerite not developed. Compound eyes hemispherical, adpressed to head. Antenna relatively short and stout, 1.0–1.3 times as long as head width, segment III the longest, bearing apical disc-shaped sensorium (Figure 2E); segments IV, VI, VIII, and IX, each with a single subapical rhinarium. Clypeus flattened, in ventral view heart-shaped, in lateral view partly hidden by genae. Rostrum 0.4–0.5 times as long as head width, in lateral view, the two apical segments visible. Pronotum transversely ribbon-shaped, bearing each a shallow sublateral and lateral pit on either side. Propleurites (Figure 2F) about as high as wide, irregularly trapezoidal; suture with both dorsal branches developed; proepisternum narrower than proepimeron. Parapteron and tegula oval, subequal in size. Metapostnotum with shallow tubercle. Mesosternum (Figure 2G) with anterior margin weakly concave, without median incision; distance between lateral edges of katepisternum, in ventral view, narrower than head width; pleurosternal suture not visible; katepisternum indistinct, only weakly produced anteriorly; basisternum very indistinctly triangular; in ventral view, tubercle of trochantin anteriad of mesocoxa weakly developed; precoxale forming obtuse angle. Metacoxa with moderately large spur-shaped meracanthus. Metafemur (Figure 2H) with three subapical bristles on the exterior face. Metatibia 0.7–0.8 times as long as head width, lacking genual spine, bearing a posteriorly open crown of 6–8 weakly or hardly sclerotized apical spurs (Figure 2I). Metabasitarsus bearing two or one outer weakly or hardly sclerotized lateral spur. Forewing (Figure 3 and Figure 4D) oblong-oval, hardly widening to apical quarter where the wing is widest, 2.7–3.2 times as long as head width, 2.4–2.5 times as long as wide; vein C+Sc weakly curved; costal break developed, pterostigma long, ending above bifurcation of M; vein R slightly shorter than M+Cu; veins Rs and M_1+2_ not fused or connected by cross-vein; vein M much longer than its branches; cells m_1_ and cu_1_ moderately large; anal break close to apex of vein Cu_1b_; surface spinules (Figure 3B,D) present in all cells, leaving spinule-free stripes along the veins; spinules in males are sparser and leaving wider free stripes along the veins; radular spinules present in cells m_1_, m_2_ and cu_1_ forming ill-defined areas along the wing margin. Hindwing about four fifths of forewing length; costal setae not arranged in groups; vein R+M+Cu indistinctly bifurcating into veins R and M, base of Cu indistinct. Male proctiger (Figure 4E and Figure 5A,D) narrowly tubular, widest in basal third, from there narrowing to apex. Paramere, in lateral view (Figure 4E and Figure 5A,D), lamellar, shorter than proctiger. Distal segment of aedeagus (Figure 4E and Figure 5G,H) lacking lateral processes, bearing a moderately sized to large apical inflation which is incised dorso-basally. Female terminalia (Figure 5I,J) long, cuneate. Circumanal ring consisting of a single row of very narrow pores. Dorsal and ventral valvulae straight, simple, the latter with a subapical ridge; lateral valvula membranous, narrowly rounded caudally. Fifth instar immature. Body (Figure 6A) flattened, in dorsal view subcircular, 1.0–1.2 times as long as wide across wing pads. Antenna 7-segmented with a single subapical rhinarium each on segments III and V, and two on segment VII. Cephalothorax sparsely beset with short to moderately long funnel-shaped setae. Eye with a moderately long funnel-shaped seta. Meso and metanotum with small tergites and sparse moderately long funnel-shaped setae. Meso and metatibiae with around four capitate and funnel-shaped setae; tarsal arolium triangular with pedicel and unguitractor; pedicel about as long as claws. Forewing pad (Figure 6B) small, oval; beset with long and short marginal, and short dorsal funnel-shaped setae (Figure 6C). Hindwing pad with a few moderately long marginal and short dorsal funnel-shaped setae. Caudal plate evenly rounded posteriorly, with 16–20 long marginal capitate setae (Figure 6E), and around 60 short dorsal funnel-shaped setae. Circumanal ring ventral, small, distance from its hind margin to abdominal apex about 2.5 times maximum length of ring; outer ring composed of a single row of very elongate, narrow pores.

Comments. *Primascena* differs from all other aphalaroidine genera in weakly or hardly sclerotized apical metatibial spurs (versus strongly sclerotized). From *Baccharopelma*, *Ehrendorferiana*, *Panisopelama*, and *Russelliana*, which all lack metabasitarsal spurs, it differs in the presence of one (extant species) or two spurs (fossil species). From *Aphalaroida* it differs in the lack of glandular setae on the body, and from *Connectopelma* in the completely separated veins Rs and M of the forewing (versus connected by r-m crossvein or partially fused). *Freysuila*, *Sphinia*, and *Zonopelma* possess a trapezoidal vertex which is subrectangular in *Primascena*, and vertex is flat in *Pachyparia*, *Prosopidopsylla*, and *Yangus*, while it is weakly bulged anteriorly in *Primascena*.

#### 3.2.3. Key to Species of Adult Primascena

1. Antennal segment III more than twice as long as segment IV (Figure 4A). Metabasitarsus with two spurs. Pterostigma of forewing (Figure 4D) narrower than adjacent cell r_1_, at base. Paramere, in lateral view, rectangular (Figure 4E). Apical inflation of distal aedeagal segment moderately sized (Figure 4E)    *Primascena subita*.

- Antennal segment III less than twice as long as segment IV (Figure 2A,B). Metabasitarsus with one outer spur. Pterostigma of forewing (Figure 3) wider than adjacent cell r_1_, at base. Paramere, in lateral view, oval (Figure 5A,C,D,F). Apical inflation of distal aedeagal segment large (Figure 5G,H)    2

2. Genal processes dark brown or black (Figure 2C). Forewing 3.0 or more times as long as head width; m_1_ cell value ≤ 1.4. Paramere, in lateral view, slightly angular apically (Figure 5A); inner face with relatively large process proximal of the middle (Figure 5B,C). Apical inflation of distal aedeagal segment relatively angular (Figure 5G). Dorsal margin of female proctiger, in lateral view, weakly sinuate (Figure 5I). Probable host plant *Ruprechtia laxiflora*    *Primascena empsycha*.

- Genal processes light (Figure 2C). Forewing shorter than 3.0 times head width; m_1_ cell value ≥ 1.5. Paramere, in lateral view, rounded apically (Figure 5D); inner face with relatively small process distal of the middle (Figure 5E,F). Apical inflation of distal aedeagal segment relatively rounded (Figure 5H). Dorsal margin of female proctiger, in lateral view, almost straight (Figure 5J). Host plant *Ruprechtia exploratricis*    *Primascena ruprechtiae*.

#### 3.2.4. *Primascena empsycha* New Species (Figure 2A–C, Figure 3A,B and Figure 5A–C,G,I)

LSID: urn:lsid:zoobank.org:act:C15FF427-901D-4F1D-99D5-5363A732750F

Diagnosis. Adults. Genal processes dark brown or black. Antennal segment III less than twice as long as segment IV. Metabasitarsus with one outer spur. Forewing 3.0 or more times as long as head width; pterostigma wider than adjacent cell r_1_, at base m_1_ cell value ≤ 1.4. Paramere, in lateral view, narrowly oval, slightly angular apically; inner face with relatively large process proximal of the middle. Apical inflation of distal aedeagal segment large, relatively angular. Dorsal margin of female proctiger, in lateral view, weakly sinuate. Immatures unknown. 

Additional characters. Adults. Coloration. Whitish. Vertex dark brown or almost black sometimes with small white dot laterally on either side; genal processes dark dorsally. Antenna with apices of segments III, IV, VI, and VIII, and entire segments IX and X dark brown or black. Tip of rostrum black. Conspicuous dark brown or almost black pattern covering entire pronotum except for a yellow longitudinal narrow streak in the middle and a sublateral white spot on either side; also dark entire mesopraescutum, a wide line along fore margin and five long, broad longitudinal stripes on mesoscutum. Forewing straw-colored. Structure. Genal processes 0.6–0.7 times as long as vertex along mid-line. Antenna 1.0–1.1 times as long as head width; segment III with 2–3 apical disc-like sensoria; rhinaria on segments IV, VIII, and IX lacking marginal spines, segment VI with a proximal comb of two spines; relative length of flagellar segments as 1.0:0.6:0.3:0.6:0.4:0.7:0.3:0.5; relative length of segment X and terminal setae as 1.0:1.1:1.2. Metatibia with 7–8 weakly or not sclerotized apical spurs; metabasitarsus with one outer spur. Forewing (Figure 3A,B), 3.0–3.2 times as long as head width; pterostigma, at base, wider than adjacent part of cell r_1_; cell m_1_ value = 2.4, cell cu_1_ value = 1.2–1.4. Terminalia as in Figure 5A–C,G,I. Male proctiger 0.5 times as long as head width; beset with long setae except for antero-basal third. Male subgenital plate elongate, beset with long setae in apical two thirds. Paramere, in lateral view, narrowly oval, slightly angular apically; outer face with long setae in apical half; inner face with large tubercle in the middle bearing an inward and slightly backward directed sclerotized tooth. Proximal segment of aedeagus strongly curved in basal third, straight in apical two thirds; distal segment straight in basal half, forming large reniform inflation in apical half which is incised dorso-basally; sclerotized end tube long and straight. Female terminalia, in lateral view, cuneate. Proctiger 0.9 times as long as head width; dorsal margin, in lateral view, almost straight; apex blunt; irregularly beset with moderately long setae in basal half, very long setae in the third quarter from base, and short setae in apical quarter; circumanal ring 0.3 times as long as proctiger. Subgenital plate, in lateral view, irregularly cuneate, 0.8 times as long proctiger; ventral margin weakly concave; apex rounded, ending in small point. Measurements. Body length (1 ♂, 5 ♀): ♂ 1.4 mm, ♀ 1.7–1.9 mm. Morphological structures (1 ♂, 1 ♀): head width ♂ 0.40 mm, ♀ 0.46 mm; antenna length ♂ 0.44 mm, ♀ 0.46 mm; forewing length ♂ 1.20 mm, ♀ 1.46 mm; ♂ proctiger length 0.18 mm; paramere length 0.14 mm; length of distal aedeagus segment 0.14 mm; ♀ proctiger length 0.42 mm. Fifth instar immature unknown.

Etymology. From the Ancient Greek adjective ἔμψυχος = animate, living (literally, those who possess a living soul), referring to the fact that this is an extant species.

Host plant. Adults were collected on *Ruprechtia laxiflora* Meisn. (Polygonaceae) which is a likely host.

Biology. Unknown.

Distribution. Brazil (Mato Grosso do Sul).

Type material. Holotype ♂: Brazil, Mato Grosso do Sul, Dourados, Embrapa campus, S22.2738° W54.8182°, 400 m, 8.x.2021, park vegetation, *Ruprechtia laxiflora* (D. Burckhardt & D.L. Queiroz) #459(13) (UFPR, dry mounted). Paratypes: 1 ♂, 6 ♀, same data as holotype but (NHMB, UFPR, dry and slide mounted, in 70% ethanol).

#### 3.2.5. *Primascena ruprechtiae* New Species (Figure 2D–I, Figure 3C,D, Figure 5D–F,H,J and Figure 6)

LSID: urn:lsid:zoobank.org:pub:96BB823E-9465-4734-83C9-5CFB221ADB15

Diagnosis. Adults. Genal processes dark light. Antennal segment III less than twice as long as segment IV. Metabasitarsus with one outer spur. Forewing shorter than 3.0 head width; pterostigma wider than adjacent cell r_1_, at base; m_1_ cell value ≥ 1.5. Paramere, in lateral view, narrowly oval, rounded apically; inner face with relatively small process distal of the middle. Apical inflation of distal aedeagal segment large, relatively rounded. Dorsal margin of female proctiger, in lateral view, almost straight. Immatures. Body 1.0–1.2 times as long as wide. Caudal plate with 16–20 long marginal capitate setae, and around 60 short dorsal funnel-shaped setae.

Additional characters. Adults. Coloration. Whitish. Vertex dark brown or almost black with a sublateral white stripe on either side; genal processes light. Antenna with apices of segments III, IV, VI, and VIII, and entire segments IX and X dark brown or black. Tip of rostrum black. Conspicuous dark brown or almost black pattern covering entire pronotum except for on each a lateral and sublateral white spot on either side, most of the mesopraescutum dark brown or black except for a white lateral spot on either side, and a narrow line along fore margin and five short, narrow longitudinal stripes on mesoscutum. Forewing weakly yellowish. Structure. Genal processes 0.4–0.6 times as long as vertex along mid-line. Antenna 1.0–1.3 times as long as head width; segment III with a subapical and an apical disc-like sensorium and two apical peg-like sensoria (Figure 2E); rhinaria on segments IV, VIII, and IX lacking marginal spines, segment VI with a proximal comb of five spines; relative length of flagellar segments as 1.0:0.6:0.5:0.6:0.5:0.7:0.4:0.4; relative length of segment X and terminal setae as 1.0:2.3:1.8. Metatibia with 6–8 weakly or not sclerotized apical spurs; metabasitarsus with one outer spur. Forewing (Figure 3C,D), 2.7–2.9 times as long as head width; pterostigma, at base, wider than adjacent part of cell r_1_; cell m_1_ value = 2.4–2.5, cell cu_1_ value = 1.5–1.7. Terminalia as in Figure 5D–F,H,J. Male proctiger 0.4 times as long as head width; beset with long setae except for antero-basal third. Male subgenital plate elongate, beset with long setae in apical two thirds. Paramere, in lateral view, narrowly oval, rounded apically; outer face with long setae in apical half; inner face with large tubercle in the middle bearing an inward and slightly backward directed sclerotized tooth. Proximal segment of aedeagus strongly curved in basal third, straight in apical two thirds; distal segment straight in basal half, forming large reniform inflation in apical half which is incised dorso-basally; sclerotized end tube long and straight. Female terminalia, in lateral view, cuneate. Proctiger 0.8–0.9 times as long as head width; dorsal margin, in lateral view, almost straight; apex blunt; irregularly beset with moderately long setae in basal half, very long setae in the third quarter from base, and short setae in apical quarter; circumanal ring 0.3–0.4 times as long as proctiger. Subgenital plate, in lateral view, irregularly cuneate, 0.6–0.7 times as long as proctiger; ventral margin weakly concave; apex rounded, ending in small point. Measurements. Body length (10 ♂, 10 ♀): ♂ 1.4–1.7 mm, ♀ 1.5–1.7 mm. Morphological structures (2 ♂, 2 ♀): head width ♂ 0.36–0.38 mm, ♀ 0.42–0.44 mm; antenna length ♂ 0.46–0.48 mm, ♀ 0.42–0.46 mm; forewing length ♂ 0.98–1.06 mm, ♀ 1.20–1.26 mm; ♂ proctiger length 0.14 mm; paramere length 0.10 mm; length of distal aedeagus segment 0.10–0.12 mm; ♀ proctiger length 0.34–0.38 mm. Fifth instar immature. Coloration. Whitish. Cephalothorax and thoracic abdominal tergites very light brown with irregular slightly darker brown patches. Antenna whitish with segments I, II, and VII brown; apex of segment VII dark brown. Tips of tarsi brown. Forewing pad light brown at base and apical half, otherwise dark brown; hindwing pad dark brown with small area at base light brown. Caudal plate dorsally with broad brown margin, an irregular, moderately wide longitudinal whitish stripe in the middle and irregularly dark brown otherwise; ventrally similar but with more expanded dark pattern. Structure. Body (Figure 6A) 1.0–1.2 times as long as wide across wing pads. Antenna 0.7–0.9 times as long as forewing pad; segments III and V each with a short capitate seta about half or two-thirds as long as diameter of respective segment. Mesotibia with two long funnel-shape setae and two short capitate setae; metatibia with four short to moderately long capitate setae. Caudal plate 0.5–0.6 times as long as wide. Measurements (six specimens). Body length 0.78–1.00 mm; antenna length 0.28–0.32 mm.

Etymology. Named after its host genus *Ruprechtia*.

Host plant. *Ruprechtia exploratricis* Sandwith (Polygonaceae).

Biology. Immatures are free-living and develop on the lower leaf face (Figure 6F). Immatures often are close to the larger veins (Figure 6G).

Distribution. Brazil (Mato Grosso do Sul).

Type material. Holotype ♂: Brazil, Mato Grosso do Sul, Corumbá, Polícia militar ambiental, S19.0121° W57.6859°, 110 m, 12–16.x.2021, degraded vegetation near river, *Ruprechtia exploratricis* (D. Burckhardt & D.L. Queiroz) #470(2) (UFPR, dry mounted). Paratypes: 75 ♂, 87 ♀, 55 immatures, 17 skins, 20 egg, same data as holotype but (NHMB, UFPR, dry and slide mounted, in 70% ethanol); 4 ♂, 4 ♀, same but Assentamento Taquaral, S19.1235° W57.6862°, 220 m, 17.x.2021, deciduous forest, *Ruprechtia exploratricis*, #478(2) (NHMB, in 70% ethanol); 4 ♂, 25 ♀, 5 skins, same but Corumbá, Cemitério Nelson Chamma, S19.0199° W57.6956°, 130 m, 17.x.2021, park vegetation, *Ruprechtia exploratricis*, #479(1) (NHMB, in 70% ethanol).

#### 3.2.6. *Primascena subita* Klimaszewsi, 1998 [13] (Figure 4)

Diagnosis. Adults. Antennal segment III more than twice as long as segment IV. Metabasitarsus with two spurs. Forewing with pterostigma narrower than adjacent cell r_1_, at base; m_1_ cell value = 1.5. Paramere, in lateral view, rectangular. Apical inflation of distal aedeagal segment moderately sized. Female and immatures unknown.

Redescription. Coloration. Body and forewing lacking distinct dark pattern. Structure. Head (Figure 4A,B) severely damaged; genal processes developed; compound eyes semicircular, adpressed to head. Clypeus and rostrum missing. Antenna 1.1 times as long as head width; relative length of flagellar segments as 1.0:0.4:0.4:0.5:0.4:0.4:0.4:0.4; relative length of segment X and terminal setae as 1.0:2.3:1.8. Thorax strongly damaged and incomplete. Metacoxa with spur-shaped meracanthus (Figure 4C); metatibia 0.8 times as long as head width, bearing a posteriorly open crown of five evenly spaced, weakly sclerotized apical spurs (Figure 4C); metabasitarsus with two spurs. Forewing (Figure 4D) 3.8 times as long as head width, 2.6 times as long as wide; with pterostigma narrower than adjacent cell r_1_, at base; m_1_ cell value = 1.5, cu_1_ cell value = 3.1. Paramere (Figure 4E), in lateral view, rectangular. Apical inflation of distal aedeagal segment moderately sized, incised at the base dorsally. Female and immatures unknown. Measurements (1 ♂). Head width 0.40 mm; antenna length 0.43 mm; forewing length 1.50 mm; proctiger length 0.15 mm; paramere length 0.10 mm; length of distal aedeagus segment 0.13 mm.

Etymology. From the Latin adjective subitus = sudden, unexpected [13].

Host plant and biology. Unknown.

Distribution. Amber fossil from the Dominican Republic.

Comments. Klimaszewski [13] described the clypeus as “bent downwards” and figured it to be a tubular structure perpendicular to the ventral head face. Klimaszewski’s drawing of the head is difficult to interpret but we think that the structure in question could be one of the genal processes (Figure 5A,B). Our measurements also differ slightly from those by Klimaszewski [13] probably because they were taken at different angles.

Type material. Holotype ♂: Dominican Republic, North Mines, Miocene amber, DR-14-137 (not DR-14-67 as indicated by Klimaszewski [13]) (AMNH).

## 4. Discussion

The psyllid fauna of Dominican amber is diverse at both the family and genus level and is similar to today’s fauna [10,12]. Burckhardt, et al. [14] reported 19 described species from Dominican amber. Six of these were originally described in extant genera, while another six species were transferred from fossil genera to recent genera due to synonymization. This large proportion of species where the systematic affinity had to be revised (almost one-third!) is a consequence of inaccurate observations and the general poor knowledge of neotropic psyllids. While six of the remaining seven fossil genera listed by Burckhardt, et al. [14] are currently known only from fossils, represented by a single species based on a single specimen, one genus, *Primascena*, also includes two extant species which are described here. The cladistic analysis strongly supports the monophyly of *P. subita* with *P. empsycha* and *P. ruprechtiae*, and confirms the monophyly of nine other genera of Aphalaroidinae (Figure 1), hence supporting the current classification of the subfamily [4,5]. While *Pachyparia* and *Yangus* are represented in the analysis by only one species, *Connectopelma* (in node 8) is paraphyletic with respect to *Panisopelma*, though the position of *Panisolepma* within *Connectopelma* is only weakly supported.

*Primascena* is sister to all but *Aphalaroida*, though with little support (Figure 1). Weak is also the support for nodes 3–5. Node 6, which includes eight genera, is strongly supported by five synapomorphies and two homoplasies with a Bremer support = 2. Within node 6, the relationships between genera are, again, only weakly supported, with the exception of *Russelliana* + *Sphinia*. While there are no other cladistic analyses examining the relationships of all recognized genera of Aphalaroidinae, Burckhardt [16] discussed possible relationships among some of the genera within the subfamily and suggested the following grouping: ((*Baccharopelma*, *Connectopelma*), (*Zonopelma*, (*Panisopelma*, (*Russelliana*, (*Sphinia*, (*Aphalaroida*, *Prosopidopsylla*)))))). The considerable difference between the two sets of phylogenetic relationships is due to the fact that the latter is derived from a discussion of characters and does not result from a parsimony analysis. Further studies should be conducted, e.g., by including more species, in particular of *Pachyparia* and *Yangus*, represented in the analysis by only one species each. *Pachyparia* is currently monotypic and *Yangus* is known only from three described Asian species. Several undescribed Afrotropical species of both genera are represented in the collection of the NHMB. In addition to morphological characters, molecular data should also be analyzed to test the present phylogeny.

Psyllids exhibit interesting host plant patterns. They are reasonably host-specific, and related psyllid species often develop on related hosts [2,3,4,6,20]. Aphalaroidinae is atypical in this respect. It comprises the only polyphagous psyllid species apart from the few *Bactericera* species [40,41]. The host range of Aphalaroidinae, without those of the polyphagous *Russelliana solanicola* Tuthill, is also surprisingly wide; the hosts belong to 12 plant families of ten orders and include conifers (Cupressaceae) and several groups of eudicots (Table 1). Four genera are entirely associated and two are partially associated with Fabaceae, the family with the largest number of associated psyllid genera. It is mostly confined to the Psyllidae [20], of which it hosts members of seven of the nine recognized subfamilies. The extant species of *Primascena* are associated with *Ruprechtia* (Polygonaceae: Eriogonoideae). This family serves as host to 69 psylloid species [5,42]. Most of these hosts are members of the subfamily Polygonoideae, which harbors Aphalaridae (27 *Aphalara* spp.), Liviidae (9 *Eremopsylloides* spp., 13 *Pachypsylloides* spp., 10 *Shaerqia* spp.), and Triozidae (5 *Bactericera* spp., 2 *Trioza* spp.). Only three species of Aphalaroidinae (Psyllidae) develop on members of the subfamily Eriogonoideae: two *Primascena* spp. on *Ruprechtia* and one *Russelliana* species on *Chorizanthe*. The majority of immature Aphalaroidinae, including *Primascena*, are free-living. Gall-inducing taxa are all species of *Baccharopelma* and some species of *Connectopelma*.

The Aphalaroidinae is predominantly American, with the exception of *Pachaparia* and *Yangus*, which are restricted to the Old World (Figure 1, Table 1). The former is known from Africa and the Arabian Peninsula while the latter has been reported from the Oriental Region [5] and is represented by undescribed species from Africa (unpublished NHMB data). *Aphalaroida* is entirely North American [43] and *Freysuila* has two North American and one South American species [44]. The other nine genera are confined to the Neotropical region [9,16,22,45]. *Russelliana* is by far the largest genus in the subfamily and has also the widest distribution. It ranges from the Chilean Far South to Peru, with a few species also occurring in Brazil [46]. It is a characteristic element of Mediterranean sclerophyll scrub. Similar habitats are used by species of the genera *Baccharopelma*, *Ehrendorferiana*, *Panisopelma*, and *Sphinia*, but they are geographically less widespread than *Russelliana*. The two species of *Zonopelma* develop on *Misodendrum* species parasitizing *Nothofagus* and occur mostly in temperate rain forests. *Primascena*, finally, is known so far only from Dominican amber and two extant species from the Brazilian Pantanal, which live in predominantly forested habitats such as riverine or gallery forests and in seasonally semideciduous forests.

## 5. Conclusions

Homoplasy in morphological characters of adult psyllids is a major problem in phylogenetic reconstruction. This is one of the reasons why fossil psyllids are often difficult to place in a phylogeny. In general, the fauna of Dominican amber is quite similar to the extant fauna at the genus level, but in psyllids almost a third of the species described from Dominican amber are assigned to fossil-only genera. Psyllids are most species-rich in the tropics and south temperate regions, but Afrotropical and Neotropical faunas are only poorly known. To find extant members of these fossil genera is, therefore, not unlikely. *Primascena* exemplifies this. The discovery of two extant species permitted a careful morphological study of the genus and added information on the female and fifth instar immature. These new morphological data provided the base for a cladistic analysis that supported the monophyly of the fossil and extant species, and confirm its placement in the Aphalaroidinae, previously suggested by a revision of the holotype of *P. subita*.

Targeted field work is not only important for discovering previously unknown biodiversity, but also to find new host associations. While psyllid subfamilies or tribes are often restricted to a single plant family or order, the host range of Aphalaroidinae is surprisingly broad. Even though *Ruprechtia* is an unusual psyllid host, it fits the pattern found in Aphalaroidinae.

## Data Availability

Data are contained within the article or Supplementary Materials.

## Supplementary Materials

The following supporting information can be downloaded at: https://www.mdpi.com/article/10.3390/insects15060382/s1, File S1: The character matrix used for the phylogenetic analysis.
